# An Important Medico-Legal Decision

**Published:** 1882-12

**Authors:** 


### AN IMPORTANT MEDICO-LEGAL DECISION.

A decision of much importance was rendered by Judge Donohue, in the Supreme Court, recently. The question for determination was as to the effectiveness of physicians' certificates alleging insanity in cases where insanity has not been legally proved. Judge Donohue held that such certificates are not *prima facie* evidence of insanity, and that the persons who procured them are bound to support the certificates with testimony in the course of a legal inquiry as to the mental state of the alleged lunatic.

The law says that no person shall be held in a lunatic asylum upon physicians' certificates for more than five days unless the certificates shall have been approved by a judge of a court of record of the district in which the alleged lunatic resides. Before granting his approval the judge may, in his discretion, take proofs as to the alleged insanity. The usual practice of the judge is, however, to approve the certificates without delay, upon the assumption that the persons who procured them will not proceed until the mental unsoundness of the subject of the inquiry is definitely determined. There can be no good reason, therefore, that they should be held to be anything else than a warrant for temporary detention.—*Med. Record.*

